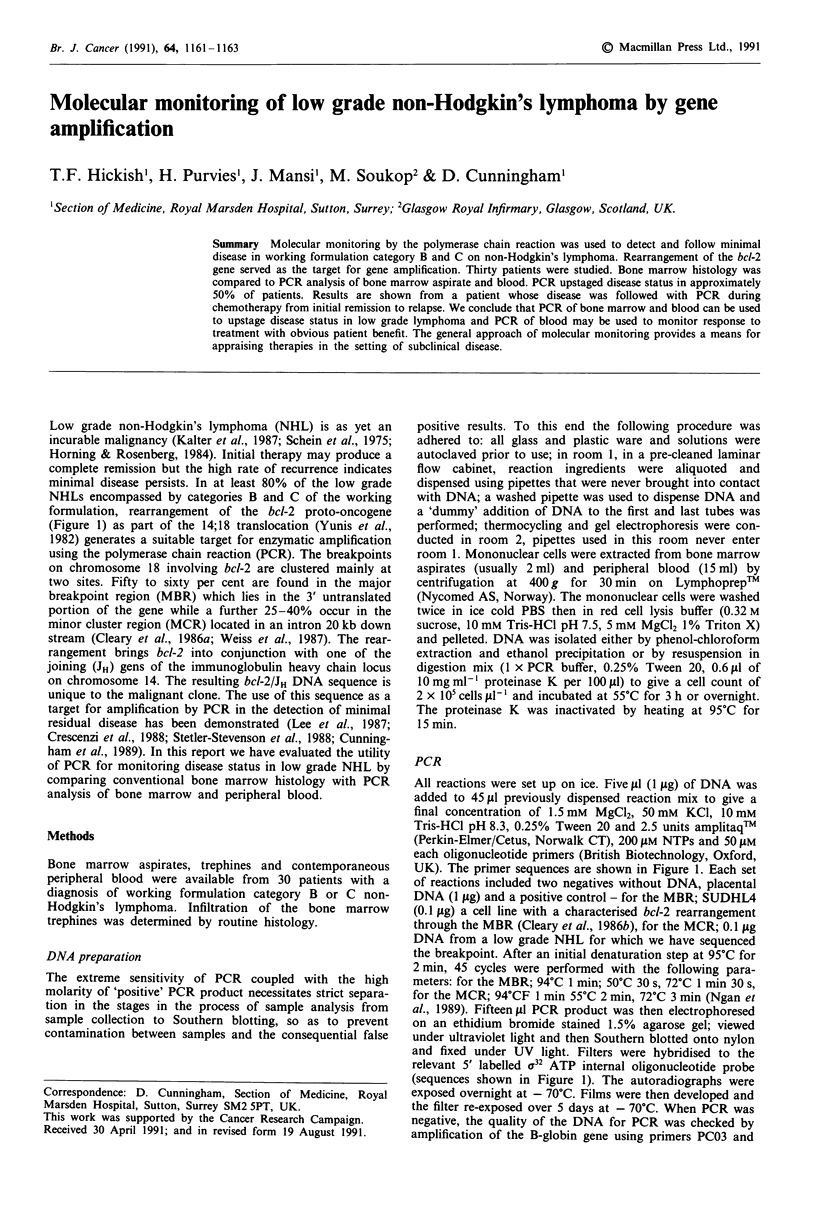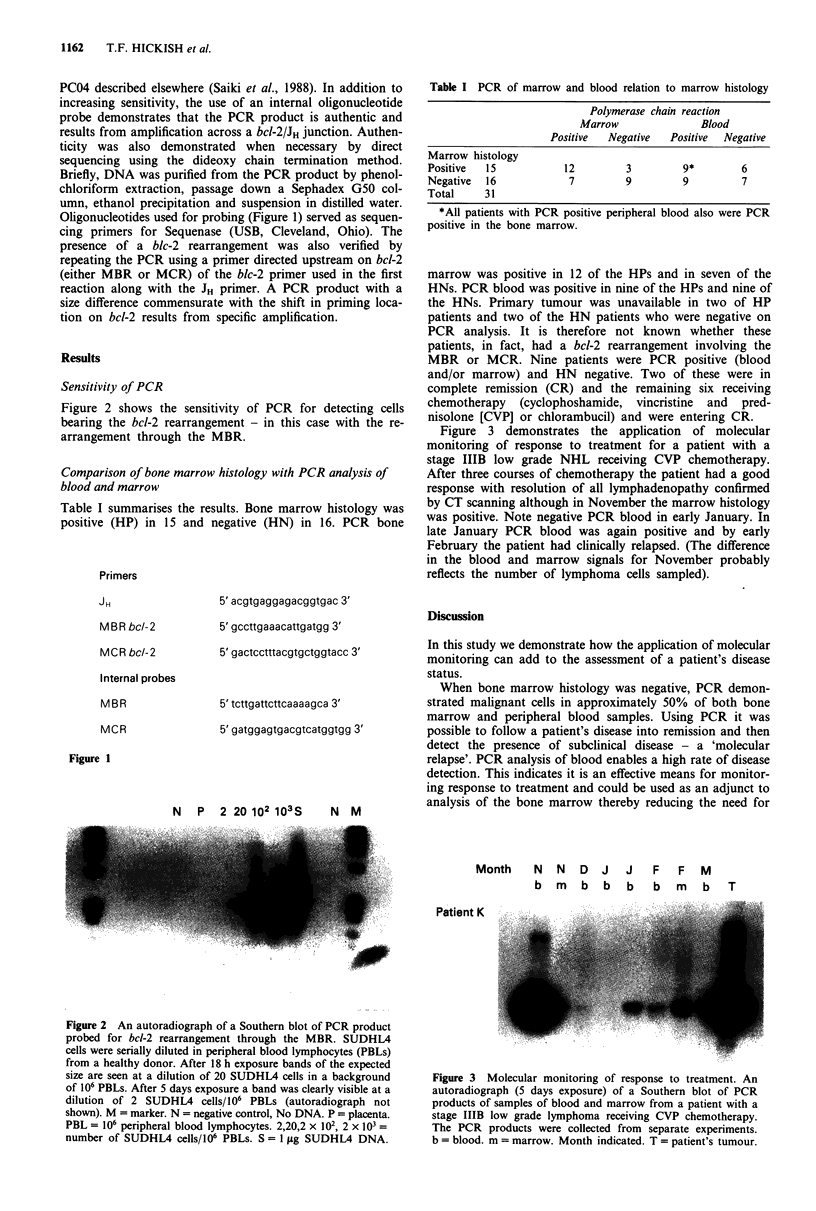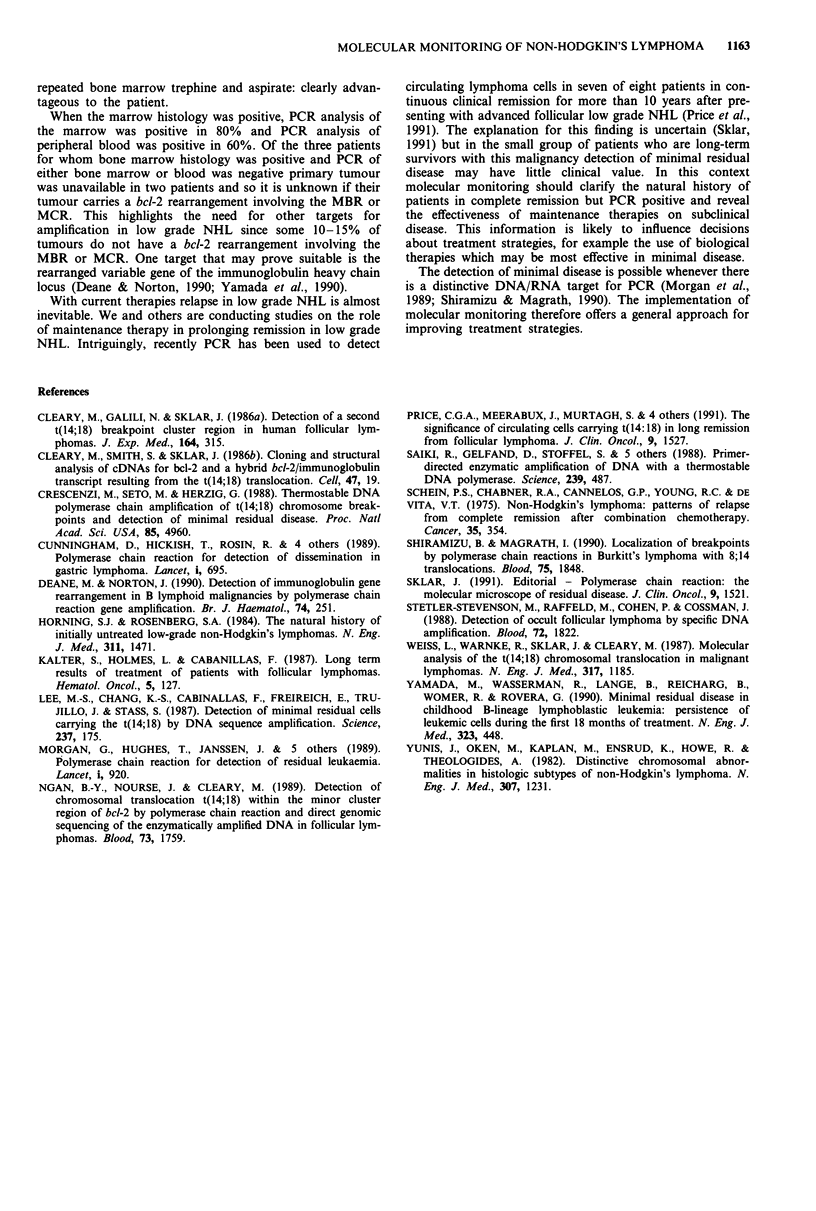# Molecular monitoring of low grade non-Hodgkin's lymphoma by gene amplification.

**DOI:** 10.1038/bjc.1991.482

**Published:** 1991-12

**Authors:** T. F. Hickish, H. Purvies, J. Mansi, M. Soukop, D. Cunningham

**Affiliations:** Section of Medicine, Royal Marsden Hospital, Sutton, Surrey, UK.

## Abstract

**Images:**


Br. J. Cancer (1991), 64, 1161–1163 © Macmillan Press Ltd., 1991

### Molecular monitoring of low grade non-Hodgkin's lymphoma by gene amplification

T.F. Hickish[1], H. Purvies[1], J. Mansi[1], M. Soukop[2] & D. Cunningham[1]

[1]*Section of Medicine, Royal Marsden Hospital, Sutton, Surrey;* [2]*Glasgow Royal Infirmary, Glasgow, Scotland, UK.*

**Summary** Molecular monitoring by the polymerase chain reaction was used to detect and follow minimal disease in working formulation category B and C on non-Hodgkin's lymphoma. Rearrangement of the *bcl*-2 gene served as the target for gene amplification. Thirty patients were studied. Bone marrow histology was compared to PCR analysis of bone marrow aspirate and blood. PCR upstaged disease status in approximately 50% of patients. Results are shown from a patient whose disease was followed with PCR during chemotherapy from initial remission to relapse. We conclude that PCR of bone marrow and blood can be used to upstage disease status in low grade lymphoma and PCR of blood may be used to monitor response to treatment with obvious patient benefit. The general approach of molecular monitoring provides a means for appraising therapies in the setting of subclinical disease.

Low grade non-Hodgkin's lymphoma (NHL) is as yet an incurable malignancy (Kalter *et al.*, 1987; Schein *et al.*, 1975; Horning & Rosenberg, 1984). Initial therapy may produce a complete remission but the high rate of recurrence indicates minimal disease persists. In at least 80% of the low grade NHLs encompassed by categories B and C of the working formulation, rearrangement of the *bcl*-2 proto-oncogene (Figure 1) as part of the 14;18 translocation (Yunis *et al.*, 1982) generates a suitable target for enzymatic amplification using the polymerase chain reaction (PCR). The breakpoints on chromosome 18 involving *bcl*-2 are clustered mainly at two sites. Fifty to sixty per cent are found in the major breakpoint region (MBR) which lies in the 3′ untranslated portion of the gene while a further 25–40% occur in the minor cluster region (MCR) located in an intron 20 kb down stream (Cleary *et al.*, 1986*a*; Weiss *et al.*, 1987). The rearrangement brings *bcl-2* into conjunction with one of the joining ($J_H$) gens of the immunoglobulin heavy chain locus on chromosome 14. The resulting *bcl*-2/$J_H$ DNA sequence is unique to the malignant clone. The use of this sequence as a target for amplification by PCR in the detection of minimal residual disease has been demonstrated (Lee *et al.*, 1987; Crescenzi *et al.*, 1988; Stetler-Stevenson *et al.*, 1988; Cunningham *et al.*, 1989). In this report we have evaluated the utility of PCR for monitoring disease status in low grade NHL by comparing conventional bone marrow histology with PCR analysis of bone marrow and peripheral blood.

### Methods

Bone marrow aspirates, trephines and contemporaneous peripheral blood were available from 30 patients with a diagnosis of working formulation category B or C non-Hodgkin's lymphoma. Infiltration of the bone marrow trephines was determined by routine histology.

### *DNA preparation*

The extreme sensitivity of PCR coupled with the high molarity of 'positive' PCR product necessitates strict separation in the stages in the process of sample analysis from sample collection to Southern blotting, so as to prevent contamination between samples and the consequential false positive results. To this end the following procedure was adhered to: all glass and plastic ware and solutions were autoclaved prior to use; in room 1, in a pre-cleaned laminar flow cabinet, reaction ingredients were aliquoted and dispensed using pipettes that were never brought into contact with DNA; a washed pipette was used to dispense DNA and a 'dummy' addition of DNA to the first and last tubes was performed; thermocycling and gel electrophoresis were conducted in room 2, pipettes used in this room never enter room 1. Mononuclear cells were extracted from bone marrow aspirates (usually 2 ml) and peripheral blood (15 ml) by centrifugation at 400 *g* for 30 min on Lymphoprep™ (Nycomed AS, Norway). The mononuclear cells were washed twice in ice cold PBS then in red cell lysis buffer (0.32 M sucrose, 10 mM Tris-HCl pH 7.5, 5 mM $MgCl_2$ 1% Triton X) and pelleted. DNA was isolated either by phenol-chloroform extraction and ethanol precipitation or by resuspension in digestion mix (1 × PCR buffer, 0.25% Tween 20, 0.6 µl of 10 mg $ml^{-1}$ proteinase K per 100 µl) to give a cell count of $2 \times 10^5$ cells $\mu l^{-1}$ and incubated at 55°C for 3 h or overnight. The proteinase K was inactivated by heating at 95°C for 15 min.

### *PCR*

All reactions were set up on ice. Five µl (1 µg) of DNA was added to 45 µl previously dispensed reaction mix to give a final concentration of 1.5 mM $MgCl_2$, 50 mM KCl, 10 mM Tris-HCl pH 8.3, 0.25% Tween 20 and 2.5 units amplitaq™ (Perkin-Elmer/Cetus, Norwalk CT), 200 µM NTPs and 50 µM each oligonucleotide primers (British Biotechnology, Oxford, UK). The primer sequences are shown in Figure 1. Each set of reactions included two negatives without DNA, placental DNA (1 µg) and a positive control – for the MBR; SUDHL4 (0.1 µg) a cell line with a characterised *bcl*-2 rearrangement through the MBR (Cleary *et al.*, 1986*b*), for the MCR; 0.1 µg DNA from a low grade NHL for which we have sequenced the breakpoint. After an initial denaturation step at 95°C for 2 min, 45 cycles were performed with the following parameters: for the MBR; 94°C 1 min; 50°C 30 s, 72°C 1 min 30 s, for the MCR; 94°CF 1 min 55°C 2 min, 72°C 3 min (Ngan *et al.*, 1989). Fifteen µl PCR product was then electrophoresed on an ethidium bromide stained 1.5% agarose gel; viewed under ultraviolet light and then Southern blotted onto nylon and fixed under UV light. Filters were hybridised to the relevant 5′ labelled $\sigma^{32}$ ATP internal oligonucleotide probe (sequences shown in Figure 1). The autoradiographs were exposed overnight at – 70°C. Films were then developed and the filter re-exposed over 5 days at – 70°C. When PCR was negative, the quality of the DNA for PCR was checked by amplification of the B-globin gene using primers PC03 and

Correspondence: D. Cunningham, Section of Medicine, Royal Marsden Hospital, Sutton, Surrey SM2 5PT, UK.
This work was supported by the Cancer Research Campaign.
Received 30 April 1991; and in revised form 19 August 1991.

PC04 described elsewhere (Saiki *et al.*, 1988). In addition to increasing sensitivity, the use of an internal oligonucleotide probe demonstrates that the PCR product is authentic and results from amplification across a *bcl*-2/$J_H$ junction. Authenticity was also demonstrated when necessary by direct sequencing using the dideoxy chain termination method. Briefly, DNA was purified from the PCR product by phenol-chloriform extraction, passage down a Sephadex G50 column, ethanol precipitation and suspension in distilled water. Oligonucleotides used for probing (Figure 1) served as sequencing primers for Sequenase (USB, Cleveland, Ohio). The presence of a *blc*-2 rearrangement was also verified by repeating the PCR using a primer directed upstream on *bcl*-2 (either MBR or MCR) of the *blc*-2 primer used in the first reaction along with the $J_H$ primer. A PCR product with a size difference commensurate with the shift in priming location on *bcl*-2 results from specific amplification.

### Results

### *Sensitivity of PCR*

Figure 2 shows the sensitivity of PCR for detecting cells bearing the *bcl*-2 rearrangement – in this case with the rearrangement through the MBR.

### *Comparison of bone marrow histology with PCR analysis of blood and marrow*

Table I summarises the results. Bone marrow histology was positive (HP) in 15 and negative (HN) in 16. PCR bone marrow was positive in 12 of the HPs and in seven of the HNs. PCR blood was positive in nine of the HPs and nine of the HNs. Primary tumour was unavailable in two of HP patients and two of the HN patients who were negative on PCR analysis. It is therefore not known whether these patients, in fact, had a *bcl*-2 rearrangement involving the MBR or MCR. Nine patients were PCR positive (blood and/or marrow) and HN negative. Two of these were in complete remission (CR) and the remaining six receiving chemotherapy (cyclophoshamide, vincristine and prednisolone [CVP] or chlorambucil) and were entering CR.

Figure 3 demonstrates the application of molecular monitoring of response to treatment for a patient with a stage IIIB low grade NHL receiving CVP chemotherapy. After three courses of chemotherapy the patient had a good response with resolution of all lymphadenopathy confirmed by CT scanning although in November the marrow histology was positive. Note negative PCR blood in early January. In late January PCR blood was again positive and by early February the patient had clinically relapsed. (The difference in the blood and marrow signals for November probably reflects the number of lymphoma cells sampled).

**Table I** PCR of marrow and blood relation to marrow histology

| | | *Polymerase chain reaction* | | | |
|---|---|---|---|---|---|
| | | *Marrow* | | *Blood* | |
| | | *Positive* | *Negative* | *Positive* | *Negative* |
| Marrow histology | | | | | |
| Positive | 15 | 12 | 3 | 9* | 6 |
| Negative | 16 | 7 | 9 | 9 | 7 |
| Total | 31 | | | | |

*All patients with PCR positive peripheral blood also were PCR positive in the bone marrow.

| Primers | |
|---|---|
| $J_H$ | 5′ acgtgaggagacggtgac 3′ |
| MBR *bcl*-2 | 5′ gccttgaaacattgatgg 3′ |
| MCR *bcl*-2 | 5′ gactcctttacgtgctggtacc 3′ |
| **Internal probes** | |
| MBR | 5′ tcttgattcttcaaaagca 3′ |
| MCR | 5′ gatggagtgacgtcatggtgg 3′ |

**Figure 1**

**Figure 2** An autoradiograph of a Southern blot of PCR product probed for *bcl*-2 rearrangement through the MBR. SUDHL4 cells were serially diluted in peripheral blood lymphocytes (PBLs) from a healthy donor. After 18 h exposure bands of the expected size are seen at a dilution of 20 SUDHL4 cells in a background of $10^6$ PBLs. After 5 days exposure a band was clearly visible at a dilution of 2 SUDHL4 cells/$10^6$ PBLs (autoradiograph not shown). M = marker. N = negative control, No DNA. P = placenta. PBL = $10^6$ peripheral blood lymphocytes. 2,20,2 × $10^2$, 2 × $10^3$ = number of SUDHL4 cells/$10^6$ PBLs. S = 1 μg SUDHL4 DNA.

### Discussion

In this study we demonstrate how the application of molecular monitoring can add to the assessment of a patient's disease status.

When bone marrow histology was negative, PCR demonstrated malignant cells in approximately 50% of both bone marrow and peripheral blood samples. Using PCR it was possible to follow a patient's disease into remission and then detect the presence of subclinical disease – a 'molecular relapse'. PCR analysis of blood enables a high rate of disease detection. This indicates it is an effective means for monitoring response to treatment and could be used as an adjunct to analysis of the bone marrow thereby reducing the need for

**Figure 3** Molecular monitoring of response to treatment. An autoradiograph (5 days exposure) of a Southern blot of PCR products of samples of blood and marrow from a patient with a stage IIIB low grade lymphoma receiving CVP chemotherapy. The PCR products were collected from separate experiments. b = blood. m = marrow. Month indicated. T = patient's tumour.

repeated bone marrow trephine and aspirate: clearly advantageous to the patient.

When the marrow histology was positive, PCR analysis of the marrow was positive in 80% and PCR analysis of peripheral blood was positive in 60%. Of the three patients for whom bone marrow histology was positive and PCR of either bone marrow or blood was negative primary tumour was unavailable in two patients and so it is unknown if their tumour carries a *bcl*-2 rearrangement involving the MBR or MCR. This highlights the need for other targets for amplification in low grade NHL since some 10–15% of tumours do not have a *bcl*-2 rearrangement involving the MBR or MCR. One target that may prove suitable is the rearranged variable gene of the immunoglobulin heavy chain locus (Deane & Norton, 1990; Yamada *et al.*, 1990).

With current therapies relapse in low grade NHL is almost inevitable. We and others are conducting studies on the role of maintenance therapy in prolonging remission in low grade NHL. Intriguingly, recently PCR has been used to detect circulating lymphoma cells in seven of eight patients in continuous clinical remission for more than 10 years after presenting with advanced follicular low grade NHL (Price *et al.*, 1991). The explanation for this finding is uncertain (Sklar, 1991) but in the small group of patients who are long-term survivors with this malignancy detection of minimal residual disease may have little clinical value. In this context molecular monitoring should clarify the natural history of patients in complete remission but PCR positive and reveal the effectiveness of maintenance therapies on subclinical disease. This information is likely to influence decisions about treatment strategies, for example the use of biological therapies which may be most effective in minimal disease.

The detection of minimal disease is possible whenever there is a distinctive DNA/RNA target for PCR (Morgan *et al.*, 1989; Shiramizu & Magrath, 1990). The implementation of molecular monitoring therefore offers a general approach for improving treatment strategies.

### References

CLEARY, M., GALILI, N. & SKLAR, J. (1986*a*). Detection of a second t(14;18) breakpoint cluster region in human follicular lymphomas. *J. Exp. Med.*, **164**, 315.

CLEARY, M., SMITH, S. & SKLAR, J. (1986*b*). Cloning and structural analysis of cDNAs for bcl-2 and a hybrid *bcl*-2/immunoglobulin transcript resulting from the t(14;18) translocation. *Cell*, **47**, 19.

CRESCENZI, M., SETO, M. & HERZIG, G. (1988). Thermostable DNA polymerase chain amplification of t(14;18) chromosome breakpoints and detection of minimal residual disease. *Proc. Natl Acad. Sci. USA*, **85**, 4960.

CUNNINGHAM, D., HICKISH, T., ROSIN, R. & 4 others (1989). Polymerase chain reaction for detection of dissemination in gastric lymphoma. *Lancet*, **i**, 695.

DEANE, M. & NORTON, J. (1990). Detection of immunoglobulin gene rearrangement in B lymphoid malignancies by polymerase chain reaction gene amplification. *Br. J. Haematol.*, **74**, 251.

HORNING, S.J. & ROSENBERG, S.A. (1984). The natural history of initially untreated low-grade non-Hodgkin's lymphomas. *N. Eng. J. Med.*, **311**, 1471.

KALTER, S., HOLMES, L. & CABANILLAS, F. (1987). Long term results of treatment of patients with follicular lymphomas. *Hematol. Oncol.*, **5**, 127.

LEE, M.-S., CHANG, K.-S., CABINALLAS, F., FREIREICH, E., TRUJILLO, J. & STASS, S. (1987). Detection of minimal residual cells carrying the t(14;18) by DNA sequence amplification. *Science*, **237**, 175.

MORGAN, G., HUGHES, T., JANSSEN, J. & 5 others (1989). Polymerase chain reaction for detection of residual leukaemia. *Lancet*, **i**, 920.

NGAN, B.-Y., NOURSE, J. & CLEARY, M. (1989). Detection of chromosomal translocation t(14;18) within the minor cluster region of *bcl*-2 by polymerase chain reaction and direct genomic sequencing of the enzymatically amplified DNA in follicular lymphomas. *Blood*, **73**, 1759.

PRICE, C.G.A., MEERABUX, J., MURTAGH, S. & 4 others (1991). The significance of circulating cells carrying t(14:18) in long remission from follicular lymphoma. *J. Clin. Oncol.*, **9**, 1527.

SAIKI, R., GELFAND, D., STOFFEL, S. & 5 others (1988). Primer-directed enzymatic amplification of DNA with a thermostable DNA polymerase. *Science*, **239**, 487.

SCHEIN, P.S., CHABNER, R.A., CANNELOS, G.P., YOUNG, R.C. & DE VITA, V.T. (1975). Non-Hodgkin's lymphoma: patterns of relapse from complete remission after combination chemotherapy. *Cancer*, **35**, 354.

SHIRAMIZU, B. & MAGRATH, I. (1990). Localization of breakpoints by polymerase chain reactions in Burkitt's lymphoma with 8;14 translocations. *Blood*, **75**, 1848.

SKLAR, J. (1991). Editorial – Polymerase chain reaction: the molecular microscope of residual disease. *J. Clin. Oncol.*, **9**, 1521.

STETLER-STEVENSON, M., RAFFELD, M., COHEN, P. & COSSMAN, J. (1988). Detection of occult follicular lymphoma by specific DNA amplification. *Blood*, **72**, 1822.

WEISS, L., WARNKE, R., SKLAR, J. & CLEARY, M. (1987). Molecular analysis of the t(14;18) chromosomal translocation in malignant lymphomas. *N. Eng. J. Med.*, **317**, 1185.

YAMADA, M., WASSERMAN, R., LANGE, B., REICHARG, B., WOMER, R. & ROVERA, G. (1990). Minimal residual disease in childhood B-lineage lymphoblastic leukemia: persistence of leukemic cells during the first 18 months of treatment. *N. Eng. J. Med.*, **323**, 448.

YUNIS, J., OKEN, M., KAPLAN, M., ENSRUD, K., HOWE, R. & THEOLOGIDES, A. (1982). Distinctive chromosomal abnormalities in histologic subtypes of non-Hodgkin's lymphoma. *N. Eng. J. Med.*, **307**, 1231.